# Health- or Environmental-Focused Text Messages to Increase Consumption of a Sustainable Diet among Young Adults: Importance of Expected Taste

**DOI:** 10.3390/foods12061297

**Published:** 2023-03-18

**Authors:** Jonathan C. Kershaw, Tze Joo Lim, Alissa A. Nolden

**Affiliations:** 1 Department of Public and Allied Health, Bowling Green State University, Bowling Green, OH 43403, USA; 2 Department of Food Science, University of Massachusetts Amherst, Amherst, MA 01003, USA

**Keywords:** plant-based protein, plant-based diets, meat-eating, moral satisfaction, subjective norms, expected taste

## Abstract

Taste is a frequently cited barrier to the greater adoption of plant-based foods, a dietary pattern associated with both health and environmental benefits. To examine the role of expected taste in promoting greater adoption of plant-based foods, we examined the impact of a text-message intervention on the expected taste of both meat- and plant-protein foods. Young adults (*n* = 159) were randomly assigned to receive either health- or environment-focused text messages twice a week for eight weeks. Study measures (pre- and post-) included dietary recalls, the expected tastiness of meat- and plant-protein images and plant-based diets, consumption intention, and person-related factors such as moral satisfaction and the subjective norms of plant-based eating and health and environmental values. Participants rating plant-protein foods tastier at baseline were more likely to report higher actual (*p* < 0.001) and intended (*p* = 0.017) consumption of plant proteins following the intervention. While text messages had a limited effect on altering the expected taste of specific plant-protein foods, the messages did elevate the expected tastiness of plant-based diets. Baseline person-related factors positively predicted changes in expected tastiness of plant-based diets. Messages promoting plant-based foods may be more effective if these foods are first perceived as tasty. Furthermore, incorporating person-related considerations into messaging strategies may improve the expected taste of plant-based foods.

## 1. Introduction

Consumption of plant-based diets (PBDs) is associated with health and environmental benefits. Specifically, plant-based diets are associated with a lower risk of numerous chronic diseases, including cardiovascular diseases, diabetes, and some cancers [1]. Furthermore, the production of plant foods has a significantly lower environmental impact compared with animal agriculture, including lower greenhouse gas emissions, water use, energy use, and eutrophication [1]. Despite the documented benefits of PBDs, the adoption of this dietary pattern has been lagging. Growing evidence reveals that PBD acceptance is limited by both product-related (intrinsic properties such as taste, texture, etc.) and person-related (e.g., the cultural value of meat, food neophobia, etc.) barriers [2,3]. Therefore, the success of consumers’ transition from animal- to plant-based products relies on addressing both product- and person-related factors. In the present work, the terms plant-based foods and plant-protein foods are both used to describe products and dishes made of legumes (beans, peas, soy, and lentils), nuts, and manufactured products primarily derived from plant proteins.

Among product-related factors, taste is considered one of the primary drivers of food purchases [4,5]. However, nearly half of Americans report that they do not like the taste of plant-based foods, and over two-thirds state they would be willing to try more plant-based foods if they tasted better [6]. Furthermore, sensory appeal has been identified as one of the primary barriers to plant-based foods and diets in a number of countries [7,8,9]. Interestingly, even though sense and taste are consisted primary motivators for consumer food choices, compared to the breadth of research examining the physical and chemical composition of plant-based materials and products, there is a relatively small body of literature examining the sensory qualities and consumer acceptance of plant-based products [10,11]. There is a need to increase the body of knowledge regarding consumers’ acceptance and perceptions of plant-based food. This information can lay the groundwork for improving the sensory experience, which is considered a key strategy for improving the acceptance of plant-based foods [6,12,13].

Consumer acceptance is primarily formed based on the intrinsic product characteristics but can be influenced by the expected experience or taste of the product [14,15]. The expected taste of a product is influenced by a number of factors, including familiarity, perceived product attributes, previous experiences with the food and related foods, and beliefs about the product [16]. For plant-protein foods, the formation of the expected taste is complex. While some plant-protein products are highly promoted to exhibit sensory properties that mimic meat, many consumers are unfamiliar with plant-protein foods or have had underwhelming or negative experiences with trying them [17]. Furthermore, plant-protein foods are often perceived by consumers to be healthy, which may also decrease their expected sensory appeal [2,18]. Due to taste being a salient barrier to the acceptance of plant-protein food, which is influenced by expected taste, we hypothesize that consumers’ willingness to adopt a plant-based diet may increase by improving the expected taste of plant-based foods.

Extrinsic food product properties, such as product claims regarding health or environmental benefits, can also influence expected taste. Although the impact of health and environmental claims on the expected taste of food products is well documented [16], studies examining the impact of such claims on plant-protein foods specifically are relatively limited. Initial evidence suggests that health, economic, and environmental information can all positively influence the consumption intention and willingness to pay for plant-protein foods, such as legumes [19,20]. However, few studies have investigated the impact of information on affective outcomes such as taste and overall liking of plant-based foods [3]. Furthermore, while there is growing evidence that person-related factors, such as values and beliefs (e.g., meat attachment) moderate the effectiveness of product claims on expected and experienced taste [21], to the best of our knowledge, studies examining the influence of person-related factors on plant-protein-food evaluation specifically are lacking.

In addition to product claims, marketing and public health campaigns represent an alternative strategy to disseminate information about plant-based diets. Due to virtually ubiquitous mobile phone ownership and ease of implementation, text messages have been explored as a potential tool to educate consumers [22]. Several studies have documented an effect of text-message education in influencing dietary behaviors [23,24,25].

We previously reported that consumers receiving biweekly text messages decreased the intention to consume animal-protein foods and increased the intention to consume some plant-based foods based on differences between baseline responses and postintervention responses eight weeks later. The text messages were more likely to increase consumption intention among participants with higher subjective norms, moral satisfaction, and self-efficacy regarding the consumption of plant-based foods [26]. Because of the salience of sensory barriers to eating plant-based foods, we hypothesized that the expected taste of plant-based foods influenced the efficacy of the text messages. Here, we conduct a secondary analysis of our previous text-message intervention to examine the role of expected taste as both a predictor of plant-based eating and an outcome of the messaging intervention. In addition, we also examine how person-related factors, including moral satisfaction, subjective norms, and health or environmental values, predicted the effect of the messages on changes in expected taste. Our primary objective is to identify strategies that can improve the expected taste of plant-protein foods. As a secondary objective, we examine the factors that impact the expected taste of meat. Findings from this study will help advance the existing literature by revealing the impact of messages on expected plant-protein taste, a significant yet understudied barrier to the greater adoption of this dietary pattern.

## 2. Materials and Methods

### 2.1. Participants

All procedures were approved by the institutional review board and informed consent was obtained prior to testing. Individuals were eligible to participate if they met the following inclusion criteria: age 18 to 26 years old, reside in the United States, responsible for more than half of their meal choices, and consume both plant-protein and animal-based foods (i.e., vegans and vegetarians were excluded). A total of 159 (102 female) participants were included in the study.

### 2.2. Text-Message Intervention

Participants were randomly assigned to either a health-focused or environment-focused text-message-intervention group. Messages between the two groups were comparable in length and structure. For example, participants in the health-message group received a message stating, “Health-friendly AND tasty? Try a plant-based burger or other meatless dish and see for yourself! Who knew being nutritious could taste so good?” while those in the environment-focused group received, “Eco-friendly AND tasty? Try a plant-based burger or other meatless dish and see for yourself! Who knew saving the planet could taste so good?!” Participants received text messages twice a week for eight consecutive weeks. Every other week, participants were asked to respond to rate how likely they were to focus more on plant-based eating the following week. The text of the messages is described elsewhere [26].

### 2.3. Questionnaires and Dietary Assessments

Participants completed the study questionnaire and dietary intake information at both baseline and the end of the study. All survey links were emailed to participants, and they could complete the survey on any device. Dietary intake was determined using the Automated Self-Administered 24-h (ASA24) Dietary Assessment Tool, version 2020, developed by the National Cancer Institute (Bethesda, MD, USA). Participants were emailed a link to complete the ASA24 on two unannounced days (one weekday and one weekend day). The ASA24 prompted participants to recall all the food they ate the previous day and helped participants estimate the amounts of those foods. Plant-protein consumption was the average total servings of legumes, nuts, seeds, and soy across the two days. Meat consumption was the average total servings of beef and pork.

Participants rated expected tastiness, perceived healthiness, and respect for the environment of various protein-rich foods. During the survey, participants viewed pictures of both meat (beef hamburger, ground beef, beef steak, grilled chicken, turkey, chicken nuggets, sausage, pork chops, and ham) and plant-protein foods (chickpeas, lentils, split peas, almonds, peanuts, pinto beans, plant-based burger, soy-based chicken nuggets, quinoa). The images displayed each food item in a form where it would be customarily consumed, such as a hamburger served on a bun, a bowl of beans, a bowl of almonds, or a cooked chicken breast. Images were presented one at a time in a randomized order and rated each construct using a 5-point scale anchored by “Not at all (tasty/healthy)”/“Produced with no respect at all for the environment” or “Very (tasty/healthy)”/Produced with very much respect for the environment”. This approach is based on previous work, which was used to assess the perceived tastiness of food products [27].

To assess perceptions of plant-based diets generally, participants responded to the question, “In my opinion, a plant-based diet is…” followed by a series of 5-point semantic differential items, including a single item for “Not tasty/Tasty” [28]. In addition, participants completed questions regarding their food consumption intention, personal values (health and green consumer values), self-efficacy, and subjective norms, as described previously [26]. Age, height, weight, gender, ethnicity, educational level, and annual household income data were also collected. Gender, income, and education were comparable between the two groups. Median age, education, and income of the sample were 23 years old, bachelor’s degree, and $50,000-$74,999 annual income, respectively. The complete demographic characteristics, survey, and content of the text messages are reported elsewhere [26].

### 2.4. Statistical Analysis

We first used a series of regression models to assess the influence of baseline taste ratings on postintervention food intake and consumption intention. Each of the four dependent variables (postintervention values for plant and meat consumption behavior and intention) was individually regressed on baseline expected taste while controlling for the baseline value of the respective dependent variable, as suggested by Cole and Maxwell for mediation analysis of half-longitudinal data [29]. Controlling for baseline consumption or intention values accounts for within-subject correlation to more effectively isolate the effect of baseline expected taste on each outcome. Message groups (health- or environment-focused) were also included in the models to identify possible differences between the two intervention groups.

We next explored whether the text-message intervention influenced taste perception by conducting paired-sample t-tests comparing baseline and postintervention responses. We also examined whether baseline person-related factors (moral satisfaction, subjective norms, and health or environmental values) were associated with changes in expected taste by regressing postintervention taste perception on baseline predictors while controlling for the baseline taste values, similar to the first analysis.

Lastly, we assessed whether gender, education, or income was associated with changes in the expected test, using independent sample t-tests and Pearson’s correlation, respectively. Due to the exploratory nature of the study, no adjustments were made for multiple comparisons. All analyses were conducted using SPSS version 26.

## 3. Results

We first explored the extent by which expected taste is associated with changes in consumption intention and behavior regarding meat and plant protein. No significant differences between text-message groups were detected, and thus, data were pooled for a single analysis. Importantly, baseline ratings of the expected taste of plant-protein foods positively predicted the impact of the text-message intervention on changes in plant-protein-consumption intention and behavior (Table 1). In other words, individuals that rated plant-protein foods as tastier at baseline were associated with a greater increase in plant-protein consumption following the intervention. Of note, neither the perceived healthiness nor the perceived environmental impact of the plant-protein-food images collected at baseline predicted changes in plant-protein-consumption behavior or intention (Table 2 and Table 3), thus highlighting the relative importance of targeting expected taste. This finding is consistent with consumer surveys identifying taste as the most important driver of food purchases [4].

While expected taste most consistently associated with changes in consumption intention and behavior, we also note associations with perceived health and environmental friendliness measures taken at baseline. Those that rated plant-based diets as healthy at baseline were more likely to decrease animal-protein-consumption intention (Table 2). Likewise, participants that rated meat images, plant-protein images, and plant-based diets as environmentally friendly were more likely to decrease animal-protein-consumption intention (Table 3). While the observation that baseline measures for the environmental friendliness of both meat and plant protein were associated with decreases in animal-protein-consumption intention may initially seem counterintuitive, it may be a reflection of the importance of sustainability to these participants. Furthermore, overall ratings for the environmental friendliness of meat went down during the study [26], suggesting that the messages effectively educated participants about the environmental impact of meat production.

**Figure 1:**
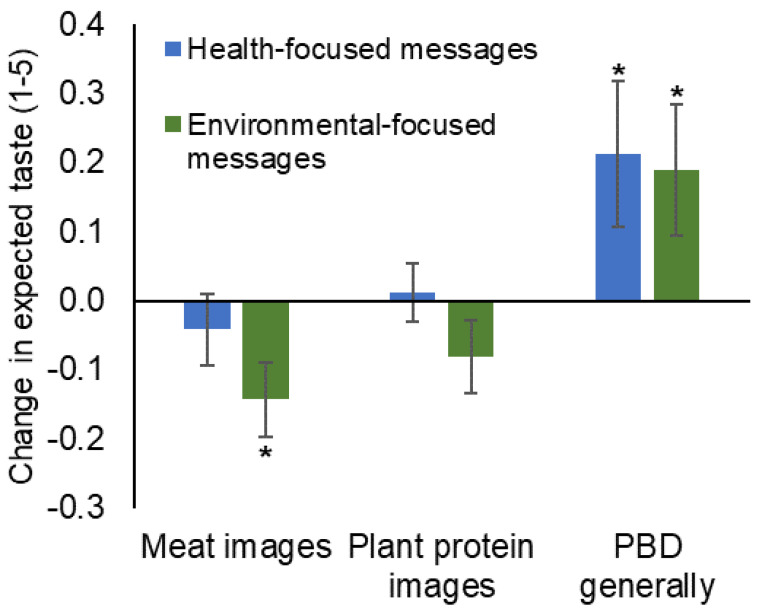
Impact of an eight-week text message intervention on expected taste, as measured by a five-point scale. Means and standard errors are represented. Asterisks indicate significant changes (postevaluation—pre-evaluation; *p* < 0.05).

Next, we analyzed the effect of text messages on the expected taste of meat- and plant-protein foods by comparing baseline and postintervention ratings within each participant. Expected taste of meat decreased, but only in the group receiving environmental-focused text messages. No differences in the expected taste of plant-protein foods were observed in either text-message group (Figure 1). However, the expected tastiness of plant-based diets did increase for both groups following the intervention (Figure 1). Although the differences were statistically significant, we acknowledge the wide variance and relatively small effect size.

We next assessed the extent by which person-related factors predicted the impact of text messages on expected taste. None of the measured constructs (moral satisfaction, subjective norm, and health and environmental values) predicted changes in expected taste of either meat- or plant-protein-food images (Table 4). However, when evaluating tastiness of PBDs, participants who derived greater moral satisfaction from plant-based eating, rated plant-based eating as more socially acceptable, and held greater health or environmental values were more likely to rate plant-based diets as tasty (Table 4).

Lastly, we explored whether gender, education, or income were associated with changes induced by the text-message intervention (Table 5). Males experienced a greater decrease in the expected tastiness of meat relative to females. However, we note that there were far fewer males than females in our study and thus we interpret this result with caution. No other associations between changes in expected taste and gender, education, or income were observed.

## 4. Discussion

Considering the challenges associated with shifting actual dietary behavior toward more sustainable patterns [30], our observation that the expected taste of plant-protein foods predicted both higher consumption intention and actual eating behavior (Table 1) positions taste as a key target for increasing the consumption of plant-protein foods. Although the text-message intervention itself did not significantly alter plant-protein intake [26], data from the present study suggest that text-messaging interventions may more effectively change dietary behavior if the food is already expected to be tasty. These data support findings from other studies that have identified the importance of the taste of plant-protein foods to increase their acceptance [3,31].

In contrast to expected plant-protein taste, expected meat taste at baseline did not significantly predict changes in meat consumption intention or behavior, irrespective of the text-message intervention (Table 1). However, we did observe that participants receiving biweekly text messages focused on environmental benefits, but not participants receiving health-focused messages, decreased in the expected tastiness of meat images (Figure 1). This observation, that environmental- (but not health-) focused messages decreased expected meat tastiness may be explained by differences in how the message interacted with participants’ values. Previous studies have demonstrated that taste evaluation improves when the values symbolized by the product (e.g., meat symbolizes social power) align with the values held by the participant [32]. Neural mechanisms may explain how values influence subjective taste: values-based labels (e.g., fair trade) improved the experienced taste pleasantness by activating neural pathways associated with reward processing [33]. In the present study, it is possible that the environmental-focused messages resonated with beliefs about the morality of meat-eating and thus impacted expected tastiness. Alternatively, the environmental messages may have impacted expected meat tastiness because they presented participants with new information: most consumers are unaware of the environmental impact of their food choices [34] and messages have the strongest impact when they provide novel information [35]. Of note, we also observed a greater impact of the messaging intervention on decreasing expected meat taste among men (Table 5), a demographic that is less willing to reduce meat consumption [36]. While acknowledging that higher initial meat-tastiness ratings may explain this observation, we suggest that taking expected taste into account may be an important consideration when designing messages to influence dietary behaviors among men. Taken together, the present data suggest that the use of environmental information may be more influential than health information when nudging consumers toward reduced meat consumption.

Although health messages did not alter the expected taste of plant-protein foods (Figure 1), previous studies suggest health is a top reason for omnivores to consider adopting a plant-based diet [37]. However, because many consumers already associate PBDs with health [3,38], the text messages may not have presented novel information, an important consideration for the effectiveness of messaging interventions [35]. Considering the general observation that perceived healthiness and expected tastiness are inversely related for most consumers [18], health messages may have a limited impact on improving expected taste. Previous studies have demonstrated that healthy (vs. hedonic) packaging cues decreased expected tastiness, but increased overall attractiveness; interestingly, the influence of packaging cues diminishes after the product is consumed [39]. Thus, while health information may improve product expectations of some product attributes, it may only have a limited impact on the expected tastiness of plant-based foods. Future studies are needed to elucidate the role of both message novelty and health information to improve the expected tastiness and consumption of plant-protein foods.

Psychological distance—defined here as the cognitive gap between imagining and experiencing something—and construal-level theory may explain why we observed an association between the intervention and the expected tastiness of a PBD but not protein-food images. Construal-level theory posits that psychologically distant concepts (e.g., PBDs in the present study) are judged based on peripheral ideas related to that concept (e.g., environmental sustainability, morality, general health, etc.); by contrast, psychologically near concepts (e.g., images of actual plant-protein foods in the present study) are judged based on their essential characteristics (e.g., taste, convenience, price, etc.) [40]. The text messages may have positively influenced the abstract idea of plant-based eating but had a smaller impact on the expectation of consuming the foods. Our finding, that the expected tastiness of a PBD was influenced conceptually but the expected taste of specific foods was not, may explain the well-documented intention–behavior gap between the stated intention to consume environmentally friendly foods and the actual purchase of them [41]. In other words, consumers may like the idea of eating plant-based foods, but when faced with the prospect of actual consumption, food-related barriers override the conceptual appeal. 

The use of abstract vs. concrete messaging strategies has practical implications for the strategic promotion of plant-based foods. We suggest that, if the messaging objective is to promote the general practice of PBDs, emphasizing ideas about health, environmental, and sustainability ideals will be more effective; alternatively, if the messaging objective is to promote a specific plant-based product, focusing on product attributes (e.g., expected taste) will be more effective. More studies are needed to confirm this relationship and consider different categories of plant-based products (e.g., meat alternatives and plant-protein foods). Regardless, we provide evidence that the concept of plant-based diets is evaluated differently than specific plant-protein foods and therefore recommend attention to related elements of study design and interpretation.

We found that higher ratings of the morality of plant-eating, increased social approval of eating plant-protein foods, and higher health or environmental values were each associated with increased expected tastiness of plant-based diets, but not plant-protein foods (Table 4). As each of these person-related factors are peripheral ideas related to the consumption of PBDs, construal-level theory [40] may again explain why associations were observed for the abstract concept of PBDs but not for the more concrete evaluation of plant-based protein images, as described in the preceding paragraph. We note that, in our previous analysis, moral satisfaction, subjective norms, and health or environmental values were generally positively associated with changes in plant-protein-consumption intention and behavior and negatively associated with animal-protein-consumption intention and behavior [26]. Others have found that the presence of ethical or moral claims, such as sustainability, fair trade, and humane treatment of animals, can improve product evaluation [42,43,44,45,46]. Additionally, manipulating participants’ sense of socially normative behavior can influence how foods are evaluated [47,48,49]. Because consumer acceptance of plant-protein foods is associated with individual characteristics such as health values and moral satisfaction [44,50], understanding how person-related factors influence actual product experiences has important implications for the promotion of plant-based products.

Several elements of the current study merit further discussion to better position our findings within a broader context. As multiweek text-message interventions have been effective in influencing dietary change [23,24], the present study represents an implementable public health strategy. While current literature examining the impact of information on plant-based-food evaluation has studied the impact of product claims [19,20], we advance the field by demonstrating that a text-message intervention can also impact the expected tastiness of plant-based diets. Furthermore, the use of food images to measure expected liking strengthens our findings, as this approach is considered a useful tool for understanding general relationships between food categories and food intake [14,15]. Importantly, we measured participants’ food intake in a free-living environment, thus increasing the external validity of the study. However, several limitations should also be considered when interpreting the findings of the current study. While the expected taste of food images can provide an indicator of acceptance of broad food categories, sensory evaluation is recommended to provide a stronger prediction of how messages impact the actual liking of specific foods [14]. Furthermore, the expected taste of the food is dependent on its presentation in the image and may vary across different presentations. Additionally, we recognize that a number of additional person-related factors influence the choice of meat- and plant-protein foods, such as concern for animal welfare, food neophobia, and meat attachment [2,37]; future work investigating the relationship between these characteristics and expected taste is important for identifying effective strategies for increasing the consumption of sustainable food choices. Cross-cultural studies investigating the impact of messaging on the expected taste of plant-based foods would further support broader efforts to increase the intake of these products.

## 5. Conclusions

In summary, these data support the importance of expected taste in promoting the acceptance and consumption of plant-protein foods. While we found limited evidence of the text-message intervention focusing on health and environmental impact on improving the expected taste of meat- or plant-protein-food images, text messages improved the expected tastiness of PBDs generally, suggesting that messages or product information may aid in improving the conceptual idea of consuming more plant-based foods. Our findings support targeting expected taste as a key strategy to improve plant-based eating.

## Figures and Tables

**Table 1:** Influence of baseline expected taste on changes in consumption intention and behavior.

Outcome (Postintervention)	Baseline Expected Taste	Std β	*p*
Animal-protein-consumption intention	Meat images	−0.044	0.535
	Plant-protein images	−0.042	0.565
	Plant-based diets	−0.043	0.558
Meat consumption	Meat images	0.062	0.402
	Plant-protein images	0.080	0.285
	Plant-based diets	0.053	0.482
Plant-protein-consumption intention	Meat images	−0.017	0.796
	Plant-protein images	**0.234**	**<0.001**
	Plant-based diets	**0.219**	**0.001**
Plant-protein consumption	Meat images	0.045	0.573
	Plant-protein images	**0.190**	**0.017**
	Plant-based diets	0.120	0.129

Because no group effects were observed, data from both groups were pooled for a single analysis. Boldface indicates *p* < 0.05.

**Table 2:** Influence of baseline perceived health on changes in consumption intention and behavior.

Outcome (Postintervention)	Baseline Perceived Health	Std β	*p*
Animal-protein-consumption intention	Meat images	−0.044	0.535
	Plant-protein images	−0.107	0.129
	Plant-based diets	**−0.234**	**0.001**
Meat consumption	Meat images	0.119	0.132
	Plant-protein images	−0.081	0.303
	Plant-based diets	0.063	0.425
Plant-protein-consumption intention	Meat images	−0.017	0.796
	Plant-protein images	0.082	0.210
	Plant-based diets	0.125	0.059
Plant-protein consumption	Meat images	0.045	0.573
	Plant-protein images	0.144	0.070
	Plant-based diets	0.086	0.276

Because no group effects were observed, data from both groups were pooled for a single analysis. Boldface indicates *p* < 0.05.

**Table 3:** Influence of baseline perceived environmental impact on changes in consumption intention and behavior.

Outcome (Postintervention)	Baseline Perceived Environmental Impact	Std β	*p*
Animal-protein-consumption intention	Meat images	**−0.167**	**0.016**
	Plant-protein images	**−0.232**	**0.001**
	Plant-based diets	**−0.151**	**0.033**
Meat consumption	Meat images	0.078	0.330
	Plant-protein images	−0.029	0.710
	Plant-based diets	−0.002	0.980
Plant-protein-consumption intention	Meat images	−0.017	0.794
	Plant-protein images	0.003	0.958
	Plant-based diets	0.114	0.082
Plant-protein consumption	Meat images	−0.065	0.414
	Plant-protein images	0.091	0.25
	Plant-based diets	0.123	0.119

Because no group effects were observed, data from both groups were pooled for a single analysis. Boldface indicates *p* < 0.05.

**Table 4:** Influence of baseline person-related factors on changes in expected taste following the text message intervention.

Expected Taste (Postintervention)	Baseline Characteristics	Std β	*p*
Images of animal protein	Moral satisfaction	−0.049	0.309
	Subjective norms	0.005	0.923
	Values (health or environment)	0.008	0.877
Images plant protein	Moral satisfaction	0.015	0.771
	Subjective norms	0.049	0.341
	Values (health or environment)	0.065	0.265
Plant-based diets generally	Moral satisfaction	**0.180**	**0.003**
	Subjective norms	**0.248**	**<0.001**
	Values (health or environment)	**0.212**	**0.001**

Because no group effects were observed, data from both groups were pooled for a single analysis. Boldface indicates *p* < 0.05.

**Table 5:** Changes in expected taste according to gender, education level, and income. For gender, Δ indicates change in females—change in nonfemales; thus, positive numbers indicate greater changes in nonfemales, based on paired *t*-tests. Pearson correlations values between changes in expected taste and education and income are also shown. Bold values indicate *p* < 0.05.

	Gender		Education		Income	
	Δ	*p*	*r*	*p*	*r*	*p*
**Meat images**	**0.17**	**0.032**	0.010	0.903	−0.123	0.122
**Plant-protein images**	−0.03	0.724	−0.052	0.511	−0.046	0.566
**Plant-based diets**	−0.07	0.632	0.119	0.136	−0.053	0.505

## Data Availability

Data available upon request.
